# Orchestration of mesenchymal plasticity and immune evasiveness *via* rewiring of the metabolic program in pancreatic ductal adenocarcinoma

**DOI:** 10.3389/fonc.2022.1005566

**Published:** 2022-11-03

**Authors:** Ari Hashimoto, Haruka Handa, Soichiro Hata, Shigeru Hashimoto

**Affiliations:** ^1^ Department of Molecular Biology, Hokkaido University Faculty of Medicine, Sapporo, Japan; ^2^ Division of Molecular Psychoimmunology, Institute for Genetic Medicine, Hokkaido University Faculty of Medicine, Sapporo, Japan

**Keywords:** EMT, inflammation, metabolic reprogramming, immune evasion, ARF6, ARID5A, IL-6

## Abstract

Pancreatic ductal adenocarcinoma (PDAC) is the most fatal cancer in humans, due to its difficulty of early detection and its high metastatic ability. The occurrence of epithelial to mesenchymal transition in preinvasive pancreatic lesions has been implicated in the early dissemination, drug resistance, and cancer stemness of PDAC. PDAC cells also have a reprogrammed metabolism, regulated by driver mutation-mediated pathways, a desmoplastic tumor microenvironment (TME), and interactions with stromal cells, including pancreatic stellate cells, fibroblasts, endothelial cells, and immune cells. Such metabolic reprogramming and its functional metabolites lead to enhanced mesenchymal plasticity, and creates an acidic and immunosuppressive TME, resulting in the augmentation of protumor immunity *via* cancer-associated inflammation. In this review, we summarize our recent understanding of how PDAC cells acquire and augment mesenchymal features *via* metabolic and immunological changes during tumor progression, and how mesenchymal malignancies induce metabolic network rewiring and facilitate an immune evasive TME. In addition, we also present our recent findings on the interesting relevance of the small G protein ADP-ribosylation factor 6-based signaling pathway driven by *KRAS*/*TP53* mutations, inflammatory amplification signals mediated by the proinflammatory cytokine interleukin 6 and RNA-binding protein ARID5A on PDAC metabolic reprogramming and immune evasion, and finally discuss potential therapeutic strategies for the quasi-mesenchymal subtype of PDAC.

## Introduction

Pancreatic ductal adenocarcinoma (PDAC) originates from epithelial cells of the exocrine pancreas, which is composed of secretory acinar cells and ductal cells (1). PDAC patients often have an unfavorable prognosis, and the 5-year overall survival rate has been reported to be only 11% in the United States (2). Only 20% of PDACs are confined to pancreatic tissue at diagnosis, approximately 30% have metastasized to regional lymph nodes, and more than 50% have disseminated to other tissues, primarily the liver and lungs (3).

Four major driver mutations have been identified in PDAC, including *KRAS*, *TP53*, *CDKN2A*, and *SMAD4/DPC4* mutations (4–6). Constitutive active mutations of *KRAS* occur in more than 90% of patients, often demonstrate oncogenic activity, and have been shown to be involved in the initiating event of PDAC tumorigenesis (6–9). In addition, oncogenic *KRAS* has been shown to promote tumor signaling through metabolic reprogramming (10) and stromal interactions (11) to facilitate tumor growth. Mutations in *TP53* also often result in oncogenic activity, and are present in up to 70% of PDACs, typically occurring at late stages of PDAC carcinogenesis, and are frequently associated with invasive and metastatic phenotypes (6, 12). Furthermore, *TP53* mutations play an important role in inducing platelet-derived growth factor (PDGF) receptor B expression, which associated with reduced disease-free survival in PDAC patients (13).

Because of the lack of effective diagnostic biomarkers for PDAC and the absence of early symptoms, the diagnosis of PDAC is often made at advanced, terminal stages. Current treatment options include surgery, if possible, or chemotherapy (gemcitabine, FOLFIRINOX [fluorouracil, leucovorin, irinotecan, and oxaliplatin], etc.), and radiation therapy, all with limited efficiency and achieving only slightly prolonged survival (14, 15). Immune checkpoint-based immunotherapies have been incorporated, albeit to a limited extent, into treatment modalities for some other cancers, but clinical trials targeting checkpoint molecules, such as CTLA4, PD-1/PD-L1, or their other cognate ligands have been unsuccessful for the treatment of PDAC. So far, there have been no successful clinical trials against PDAC, even those targeting multiple immune checkpoints (16–18).

PDAC cells also demonstrate a poor nutritional status, high levels of oxidative stress, inflammatory stress, extracellular acidosis, hypoxia, and decreased angiogenesis (15, 19, 20). Consistently, these are strong selection pressures that enable only cells that have adapted their metabolism to these hostile conditions to survive and proliferate. Notably, accumulating lines of evidence suggest that these adaptations also make PDAC cells more invasive, metastatic, stem cell-like, and resistant to therapeutic treatments (21). Consistently, several genome-wide gene expression profiling and genomic sequencing approaches to elucidate the molecular landscape of PDAC have demonstrated that the so-called basal-like (also known as quasi-mesenchymal-like or squamous) subtype is associated with a less favorable prognosis than other subtypes (22–25). Importantly, PDAC metabolite profiling and transcriptional analysis confirmed that the quasi-mesenchymal-like subtype is associated with the glycolytic subtype (26–28). This reorganization of pancreatic cancer cell metabolism opens the way for new therapeutic opportunities (20). However, the substantial heterogeneity in gene expression and metabolic characteristics, the plasticity of pancreatic cancer cells, and the pathological changes associated with their linked physicochemical and biological changes in the tumor microenvironment (TME) make PDAC a challenging disease to cure (26, 27, 29).

In this review, we summarize recent studies on how gene expression changes *via* intrinsic genetic mutations and epigenetic alterations involved in the acquisition of mesenchymal traits in PDAC cells, particularly post-transcriptional dysregulation of expression, are linked to metabolic reorganization associated with immunosuppressive TME formation during the development and malignant progression of PDAC.

Recently, PDAC has been hypothesized to be associated with two morphologically distinct precursors, i.e., pancreatic intraepithelial neoplasia (PanIN) and intrapapillary mucinous neoplasia (IPMN). PanIN can progress to invasive carcinoma in a stepwise and linear manner, which is an established mechanism of PDAC progression (30). Multiple studies have reported the sequential accumulation of PDAC driver gene mutations in PanIN, with *KRAS* mutations being the earliest known genetic alterations, being present in more than 90% of all PanINs regardless of cancer grade (31). On the other hand, the inactivation of *CDKN2A* is rare in low-grade PanIN, but has been reported to occur in more than 70% of high-grade PanIN (32). Mutations in *TP53* and *SMAD4* occur during the late stages of PanIN progression, and are almost exclusively found in high-grade PanIN and invasive PDAC. In contrast, IPMN is driven by four driver gene mutations of pancreatic tumorigenesis similar to PanIN, including early mutations in *KRAS* and late mutations in *CDKN2A*, *TP53*, and *SMAD4* (33). However, there are also two frequently altered driver genes specific to the IPMN pathway. Mutations in the oncogenic hotspot of *GNAS* are known to occur early in IPMN tumorigenesis (33–35). In addition, although inactivating mutations in *Ring finger protein 43* (*RNF43*), which encodes a ubiquitin ligase involved in WNT signaling (often with loss of heterozygosity) are also common in IPMNs (36), the precise timing of the occurrence of *RNF43* mutations in IPMN tumorigenesis has not yet been clarified to date.

In addition, we present our recent findings on the intriguing relevance of the small G protein ADP-ribosylation factor 6 (ARF6)-based signaling pathway driven by *KRAS/TP53* mutations, as well as the inflammation amplifying signals mediated by the inflammatory cytokine interleukin 6 (IL-6) and the RNA-binding protein AT-rich interactive domain 5a (Arid5a) on PDAC metabolic reprogramming and immune evasion. We will present our recent findings on the relevance of these pathways, and finally discuss potential therapeutic strategies for the quasi-mesenchymal subtype of PDAC.

## Plasticity of adult pancreatic tissues

The pancreas is an important organ responsible for metabolic control in the body, and is composed of two morphologically and functionally distinct components. The exocrine pancreas, accounting for more than 95% of total organ mass, is composed of acinar cells, which produce digestive enzymes, and ductal cells, which deliver these enzymes to the intestine. On the other hand, the endocrine islets of Langerhans consist of five different cell groups (α, β, δ, PP, and ε cells) that secrete various hormones, such as insulin and glucagon, and play crucial roles in the regulation of glucose metabolism. The exocrine and endocrine pancreas are associated with different diseases. Pancreatitis and pancreatic cancer, mostly PDAC, arise from the exocrine pancreas, whereas rare pancreatic neuroendocrine tumors arise from the endocrine islets, and diabetes is also a result of endocrine islet dysfunction (37). The mammalian pancreas has the capacity for regeneration after injury even in adults, with the acinar compartment having the highest plasticity in humans. Through epigenetic transcriptional regulation, acinar cells can dedifferentiate into an embryonic progenitor-like phenotype, and commit to either insulin^+^ β-cells (38) or ductal cells (known as acinar to ductal metaplasia [ADM]) (39, 40). ADM transdifferentiation occurs in chronic pancreatitis *via* nuclear factor-κB (NF-κB) activation, and is associated with pancreatic intraepithelial neoplasia, which is a necessary step for the generation of neoplastic precursor lesions called PanINs (Pancreatic intraepithelial neoplasia) (41–43). Thus, it has been speculated that the acinar cells of the exocrine pancreas maintain plasticity to adapt to changes in the external environment, and that their dysregulation leads to pancreatitis and pancreatic cancer.

## Heterogeneity of PDAC

To date, gene expression studies of PDAC have included comprehensive analyses focusing on subtyping of primary tumors obtained by surgical resection. Representative reports include the three-group classification by Collisson et al. (classical, quasi-mesenchymal, exocrine-like) (22), the two-group classification by Moffitt et al. (basal-like, classical) (23), and the four-group classification by Bailey et al. (squamous, immunogenic, pancreatic progenitor, and aberrantly differentiated endocrine exocrine) (24). Each of these classifications has been able to predict the prognosis of patients with resected PDAC on multivariate analysis. Notably, in about half of PDAC tumors, increased expression levels of hypoxia-associated genes were observed by RNA sequencing (RNAseq), and were substantially associated with basal-like subtypes, although there was no redundancy in the identified gene sets (44). Regarding morphology, PDACs are classified as having more or less than 40% glandular histogenesis, and are strongly associated with classical or basal subtypes, respectively (45). The squamous morphology found in more than 30% of invasive tumors has also been associated with basal-like tumors by several groups (16, 45). However, the mechanism by which PDAC diverges into various subtypes in the process of tumor evolution remains unclear. Recently, it has been reported that re-categorization of PDAC subtypes in a combined cohort of primary and metastatic tumors using single-cell RNAseq (scRNAseq) can lead to the extension of the two groups of basal-like and classical into five groups: “basal-like A”, “basal-like B”, “classic A”, “classic B”, and “hybrid” (46). These data sets, combined with cohort of patients with PDAC, enable the broad categorization of basal-like A and basal-like B into two disease subtypes, localized and metastatic disease, respectively. Thus, it is suggested that PDAC proceeds as a mixture of both expressed phenotypes, and that the behavior of the dominant phenotype and subtype is due to plasticity in both (46). The driver mutations for the classical and basal-like subtypes were shown to be biallelic loss of *SMAD4* with *GATA6* amplification, and biallelic loss of *TP53* and/or *CDKN2A* with mutant *KRAS* allele amplification, respectively, but none of the features were completely exclusive (45, 46). Therefore, whereas scRNAseq analysis of precancerous lesions to determine whether these expression phenotypes are established in PanIN has not been performed to date, the early acquisition of asymmetric driver gene mutations is itself dynamic, presumably dictating PDAC behavior, suggesting that both clonality and plasticity of PDAC cells are responsible for the histological and biological heterogeneity.

## Current diagnosis and treatment methods of pancreatic tumors

### Symptoms of PDAC and its diagnosis

Symptoms of PDAC are often vague and nonspecific, and hence it is sometimes referred to as the ‘silent killer’; in fact, 30% to 35% of patients are diagnosed with locally advanced stages and 50% to 55% with metastatic stages of disease. Biomarkers for the early detection of PDAC have not yet been identified. The most common site of this tumor is the head of the pancreas, which causes biliary obstruction, resulting in dark urine, jaundice, appetite loss, fatigue, weight loss, and exocrine pancreatic insufficiency (47).

As early symptoms of PDAC are less frequent than those of any other cancer, and a method for its early diagnosis has not been established, multidisciplinary examinations are required to detect the pancreatic tumor. The pancreas is a digestive organ that also acts as an endocrine system, and hence has abundant blood vessels. This feature makes PDAC easy to metastasize and difficult to resect. There are four clinical stages in PDAC; 1) I–II resectable (5-year survival rate, 35%–45%), 2) II–III borderline resectable (10%–15%), 3) II–III locally advanced (10%–15%), and 4) metastatic (< 5%). Pancreas computed tomography (CT) angiography with chest and pelvis CT can be used for assessment of the vascular anatomy of the pancreas. The degree of contact between the tumor and local blood vessels is classified into three levels; uninvolved, abutted, or encased. The difference between abutment and encasement is the degree of circumferential tumor-vessel involvement; existence of the tumor more than 180 degrees around the vessel implies encasement. Magnetic resonance imaging and cholangiopancreatography are also helpful to assess the possibility of metastasis in indeterminate liver lesions, and are also useful for the identification of cancers that are poorly characterized on CT imaging (47).

### Conventional treatments and ongoing notable clinical trials

**Figure 1:**
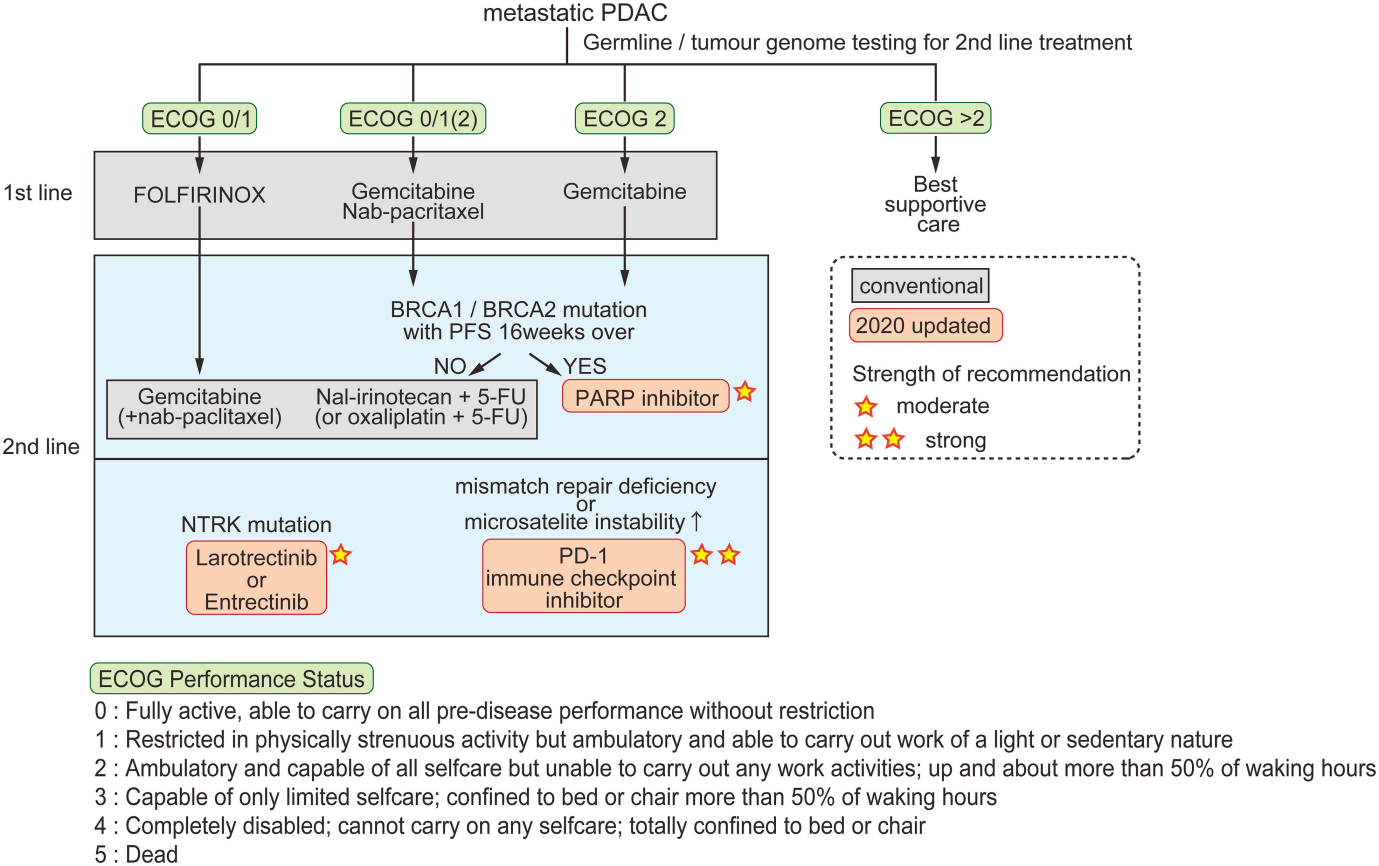
Conventional and updated guidelines for metastatic PDAC. Patients are classified by ECOG performance status for first-line chemotherapy. For second-line therapy, three options are recommended in the 2020 updated guidelines. The strength of recommendation is indicated by the number of stars.

Patients with nonresectable tumors are treated by chemotherapy according to their cancer stage and Eastern Cooperative Oncology Group (ECOG) performance status (48). Combinations of cytotoxic chemotherapies were developed in the previous decade and are still the basis of current treatments for metastatic pancreatic cancer (**Figure 1**) (49). Two multidrug regimens are now offered; FOLFIRINOX, and gemcitabine combined with nanoparticle albumin-bound paclitaxel (nab-paclitaxel). Gemcitabine alone is offered to patients with ECOG performance status 2 (within the five ECOG criteria, the groups in which patients are capable of self-care but are unable to carry out any work activities; i.e., patients are up and about > 50% of their waking hours, **Figure 1**). To classify patients eligible for either FOLFIRINOX or gemcitabine plus nab-paclitaxel as first-line drugs, Knox et al. demonstrated that a low level of GATA6, which is a characteristic of basal-like tumors, is a useful biomarker for selecting gemcitabine plus nab-paclitaxel in first-line therapy (50). The PASS-01 study analyzing the usefulness of GATA6 as a surrogate marker is now ongoing (NCT04469556).

Recent scientific advances have made incremental progress for the treatment of specific subgroups of pancreatic tumors. The American Society of Clinical Oncology guidelines updated in 2020 state three recommendations for pancreatic cancer; [1] early testing of both germline and tumor cells for microsatellite instability/mismatch repair deficiency, *BRCA* mutations, and *NTRK* gene fusions, [2] larotrectinib or entrectinib after first-line therapy for patients with tumors harboring *NTRK* fusions, and [3] continued treatment, including chemotherapy or olaparib, for patients with a germline *BRCA1* or *BRCA2* mutation who have received first-line platinum-based chemotherapy (51).

Although oncogenic *KRAS* mutations are observed in almost 90% of PDACs, a lack of drug-accessible pockets in the KRAS protein has hindered the development of their inhibitors for many years. However, X-ray crystallography identified a cryptic pocket of KRAS^G12C^ potentially useful for drug development (52, 53). A phase 1/2 clinical trial for the clinical-grade KRAS^G12C^ inhibitor AMG-510 (sotorasib) is currently ongoing (NCT03600883). Other drugs targeting mutant KRAS proteins are also being developed (54).

Clinical trials of immune checkpoint inhibitors (ICIs) against PDAC were started with great expectations, but let researchers down because of their limited efficacy compared with their efficacy against other solid tumors, including melanoma and lung cancer (16, 17). These disappointing results were attributed to the unique characteristics of PDAC, which are explained in the following sections. Given these facts, ICI treatment in combination with other types of agents to increase treatment efficacy have been widely considered for the treatment of PDAC (54).

The concept of targeting cancer metabolism has existed for almost a century, since Otto Warburg’s observation of aerobic glycolysis in cancer cells and Sidney Farber’s paper describing anti-folate-induced remission of childhood acute lymphocytic leukemia (55, 56). Their concepts were eclipsed for some time during which knowledge on oncogenes accumulated and molecular-targeting therapies showed substantial effects on patient survival. However, recent technological innovations leading to various omics analyses have clarified the connection between tumor-associated genes and metabolism (57). Mitochondria, which play various roles in cancer metabolism and malignancy, are typical targets of metabolic agents (58). The lipoate analogue CPI-613, which inhibits pyruvate dehydrogenase and α-ketoglutarate dehydrogenase and therefore disrupts mitochondrial function (59), is being evaluated in a phase III trial of metastatic PDAC (NCT03504423) (60). In this trial, both groups (with or without CPI-613) are treated with FOLFIRINOX, because it has been reported that CPI-613 enhances FOLFIRINOX cytotoxicity in some PDAC cell lines (14). Another treatment target of PDAC is autophagy, which is activated in PDAC (61). A clinical trial of combinatorial treatment of hydroxychloroquine, an inhibitor of lysosomal scavenging, a MEK inhibitor, and ICIs for PDAC patients is now ongoing (NCT04214418). As discussed here, the paradigm of targeting not only tumor cells but also the TME, including immune cells, could bring a bright future to PDAC therapy.

## Acquisition of mesenchymal plasticity of PDAC cells, and clinical implications of EMT in PDAC

### TME and mesenchymal plasticity of PDAC

A variety of stimuli, including mechanical stress, low pH, hypoxia, innate and adaptive immune responses, changes in the extracellular matrix (ECM), and treatment with antitumor drugs can activate epithelial-mesenchymal transition (EMT) in cancer cells (62). It has been shown in real clinical settings that EMT plays a role in pancreatic cancer cell dissemination to distant organs in the precancerous stage prior to and/or in parallel with primary tumor formation in PDAC (63). The fact that almost all patients who undergo complete surgical resection and are free of metastases at that time eventually die within 5 years is consistent with the early-seeding model (64–66), suggesting important roles for EMT in PDAC progression and its contribution to the poor outcome.

PDAC has been well documented to be a desmoplastic stroma consisting of a dense ECM infiltrated with heterogeneous cell populations, including immune cells, endothelial cells, and cancer-associated fibroblasts (CAFs) (67). The high density of the stroma limits oxygen supply to and diffusion in the TME, leading to the creation of a hypoxic environment. Desmoplasia is observed in the bulk of the ECM, and contains collagen, fibronectin, laminin, and hyaluronic acid. These ECM components are primarily produced by CAFs. CAFs are also involved in producing various cytokines, such as transforming growth factor β (TGF-β), IL-1, IL-6, and tumor necrosis factor, and facilitate EMT signaling pathways (68).

PDACs are characterized by hypovascular tumors in a hypoxic microenvironment, in which high interstitial fluid pressure occurs owing to desmoplasia (69). However, microvessel density (MVD) has been shown to vary considerably among PDAC tumors with its decline being associated with poor survival in inverse correlation with stromal surface area (70). The hypoxic microenvironment has broad effects on the biological behavior and malignant phenotype of PDAC, including pathological angiogenesis and metabolic reprogramming, synergistically contributing to PDAC development and therapeutic resistance. Hypoxia-inducible factors (HIFs) are essential for hypoxia-induced angiogenesis in PDAC through transcriptional activation of various angiogenic factors, such as vascular endothelial growth factor (VEGF). It has been shown that under hypoxic conditions, NF-κB activates the transcription of HIF-1α and its target gene *VEGF-A*, resulting in the increased secretion of VEGF, and enhanced angiogenesis in hypoxic pancreatic cancer cells (71). Phosphorylated signal transducer and activator of transcription 3 (STAT3) is also a hypoxia-responsive nuclear transcription factor that has been shown to act synergistically with HIF-1α to regulate angiogenesis under hypoxia in pancreatic cancer cells (72). Indeed, increased production of VEGF has been demonstrated in human PDAC cell lines and resected PDAC tumor tissues (73), showing that VEGF is produced under the control of activated HIF-1α and STAT3 under conditions of oxygen deprivation (74, 75). VEGF produced by human PDAC cell lines has functional activity to promote endothelial cell growth *in vitro*, and in large tumors in immunocompromised mouse xenograft models (76). In addition, the anti-VEGF strategy was shown to markedly reduce the growth of human PDAC cell lines orthotopically implanted into mice with a decrease in tumor MVD (77, 78). Despite these preclinical data suggesting that angiogenesis is important in PDAC, the use of anti-angiogenic agents has not been clinically successful for treating PDAC. Chronic treatment with VEGF antibodies was found to induce hypoxia and lead to increased collagen deposition, epithelial plasticity, and metastatic burden (79). These results may underly the lack of success of angiogenesis inhibitors in clinical trials of PDAC.

We previously showed that ARF6 is activated by VEGF in endothelial cells and is required for VEGF-induced tubular formation and migration. Furthermore, we have shown that ARF6 signaling is involved in choroidal neovascularization, which is a major cause of vision loss in patients with age-associated macular degeneration. We also found that ARF6 signaling is involved in VE-cadherin recycling, and may be involved in the sprouting process of angiogenesis associated with VE-cadherin-based cell-cell junctions as well as cell migration/tubular network formation activities (80). In addition, we found that high expression of the Arf6 effector AMAP1 is associated with the fibrosis of pancreatic cancer (81).

Treatment strategies for PDAC targeting angiogenesis have been pointed out as a way to normalize the tumor vasculature, such as strategies that prune immature and inefficient blood vessels, eliminate unproductive vasculature, and enable the reliable delivery of intravenous cancer drugs (82, 83). The inhibition of ARF6 signaling, which is important for pathological angiogenesis and fibrosis, may contribute to therapeutic strategies for PDAC.

Recent analyses have redefined the view that cellular senescence is the onset of the tissue remodeling that operates during normal embryonic development and tissue damage. To this end, senescent cells cease their own proliferation and recruit phagocytotic immune cells to promote tissue regeneration (84). On the other hand, it is well known that senescence is associated with cancer; in PDAC, senescence appears to produce tumor suppressive effects at the earliest stages. However, some lines of evidence indicate that senescent cells in the TME can produce a senescence-associated secretory phenotype (SASP), mediated by NF-κB and CCAAT/enhancer-binding protein-β, including the secretion of proinflammatory cytokines (IL-6 and IL-8), chemokines (monocyte chemoattractant proteins [MCPs], macrophage inflammatory proteins [MIPs], TGFβ, and granulocyte–macrophage colony-stimulating factor [GM-CSF]), and proteases (84), and play protumorigenic roles during tumor progression (85). SASPs have been shown to induce cell plasticity by stimulating cancer cell proliferation, motility, and invasion, and by generating an inflammatory TME (86). Thus, in the PDAC microenvironment, SASP may be involved in promoting EMT.

### Role of EMT in PDAC metastasis

An important aspect of the EMT program in cancer biology may be its involvement in not only facilitating cellular motility and invasiveness, but also in orchestrating the cancer stem cell state (CSCs) *via* epithelial-mesenchymal plasticity (87–89). Mechanistically, intrinsic oncogenic mutations, epigenetic gene expression conversion, and extrinsic inflammatory signals may enable highly epithelial and highly mesenchymal non-CSCs to reversibly transition to an intermediate quasi-mesenchymal state; in the case of epithelial cells, the transition is accompanied by EMT, whereas in the case of highly mesenchymal cells, it is induced by mesenchymal-epithelial transition. Presumably, similar responses might occur in normal epithelial tissue when stem cells are lost. Thus, in the invasion-metastatic cascade, the EMT program is thought to enable the seeding of cells from the primary tumor into the parenchymal layer of distant tissues, and subsequently confers stemness, giving the disseminated tumor cells the ability to form metastatic colonies (87–89).

Although it is clear that EMT is involved in tumor metastasis, the exact function of EMT in cancer is still being debated. Indeed, some studies on the effects of the EMT-transcription factors (TFs) SNAIL and TWIST in pancreatic cancer have questioned the role of EMT in metastasis. A study using PDAC model KPC (*Pdx1-cre; LSL-Kras^G12D^;Tp53^R172H^* ^/+^) mice, in which *TWIST* and *SNAIL* were independently conditionally knocked out, resulting in *Pdx1-cre; LSL-Kras^G12D^;Tp53^R172H/+^;Twist1^flox/flox^* and *Pdx1-cre; LSL-Kras^G12D^;P53^R172H/+^;Snai1^flox/flox^* mice, respectively, found that although EMT was suppressed, the deficiency of *SNAIL* or *TWIST* did not affect tumor progression, regional invasion, or dissemination. Thus, it has been argued that EMT is not required for invasive and metastatic activities of cancers. On the other hand, mice bearing abrogation of EMT-transcription factor (EMT-TF) have been shown to be correlated with chemosensitivity to gemcitabine, indicating EMT induces chemotherapy resistance in pancreatic cancer (90). Similar results have been reported in breast cancer models (91). However, other groups have shown using the same KPC mouse PDAC model that ZEB1 conditional knockout mice (*Pdx1-cre; LSL-Kras^G12D^;Tp53^R172H/+^;Zeb1^flox/flox^*) have significantly reduced PanIN and PDAC formation, and invasion and metastasis, thus clearly demonstrating a crucial role for the EMT-TF ZEB1 in the PDAC progression (92). Taken together, these studies indicate a trend toward the differential functions of EMT-TF; SNAIL and TWIST do not appear to be necessary, whereas ZEB1 conversely appears to be an important factor that is not compensated by other EMT-TFs.

## Metabolic characteristics of PDAC

### Glucose metabolism

**Figure 2:**
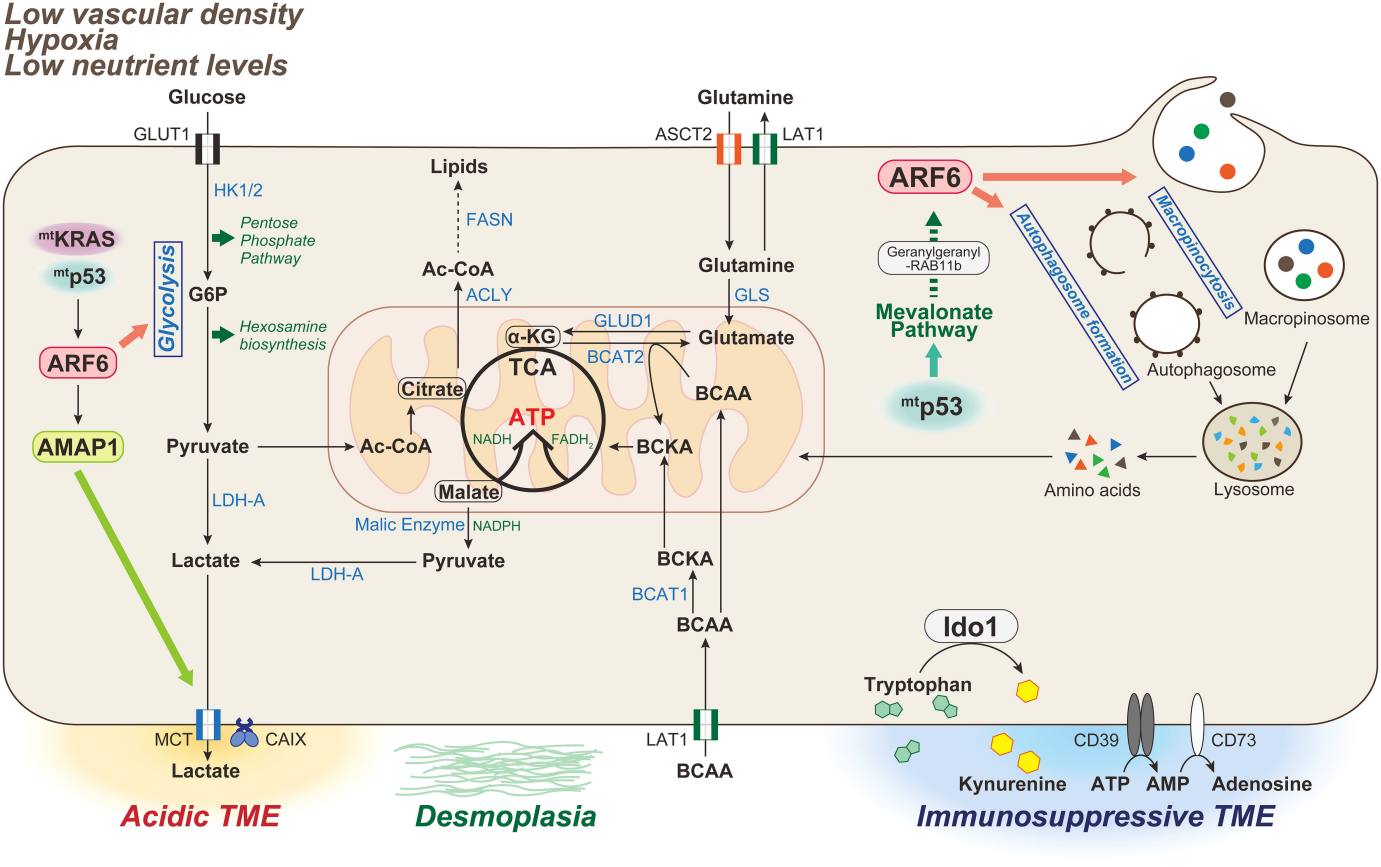
Metabolic characteristics of PDAC associated with the ARF6-based pathway. The tumor microenvironment (TME) in PDAC is characterized by low vascular density, resulting in severe hypoxia and low nutrient levels. PDAC is also characterized by a dense desmoplastic stroma. In mammals, glucose and glutamine are two of the most abundant nutrients that support cell survival and growth. Oncogenic *KRAS* mutations induce metabolic reprogramming by triggering the uptake of glucose, leading to increased glycolytic flux, carbon donation to the pentose phosphate pathway and hexosamine biosynthetic pathway, and lactate production driving acidic TME. Glutamine is also used as an energy substrate in the TCA cycle, and maintains the intracellular redox state of PDAC cells in an oncogenic *KRAS-*driven manner. Double mutations of *KRAS*/*p53* cooperatively promote the expression and activation of the ARF6-AMAP1 pathway, and ARF6 is involved in maintaining the Warburg effect to meet the abnormal nutritional and energy demands of PDAC cells, as well as those required for autophogosome and macropinosome formation. Mutant p53 promotes ARF6 activation *via* the enhanced expression of mevalonate pathway enzymes, and also intracellular trafficking of ARF6 mediated by geranylgeranylation of RAB11b. TCA, tricarboxylic acid; HK-1/2, hexokinase-1/2; G6P, glucose 6-phosphate; LDH-A, lactate dehydrogenase-A; MCT, monocarboxylate transporter; CA, carbonic anhydrase; Ac-CoA, Acetyl-CoA; ACLY, ATP-citrate lyase; FASN, fatty acid synthase; GLS, glutaminase; GLUD, glutamine dehydrogenase; BCAA, branched-chain amino acid; BCAT, branched-chain amino acid transaminase 1; IDO, indoleamine 2,3-dioxygenase.

Glucose is the principal carbon and energy source for the growth and maintenance of mammalian cells. Glucose catabolism occurs by two metabolic pathways; glycolysis and the tricarboxylic acid (TCA) cycle. These pathways not only fuel adenosine triphosphate (ATP) production, but also produce carbon intermediates that support macromolecular biosynthesis. One contribution of oncogenic *KRAS* mutations to the oncogenesis and progression of pancreatic cancer is oncogenic *KRAS* mutation-driven metabolic rewiring. Transcriptome and metabolomic analyses indicated that the activity of oncogenic *KRAS* mutations promoted the upregulation of key metabolic enzymes involved in glucose metabolism, including glycolysis, hexosamine biosynthesis leading to the synthesis of uridine diphosphate N-acetyl-glucosamine, which is a significant substrate for protein glycosylation, and the pentose phosphate pathway producing NADPH and ribose 5-phosphate, which are essential for nucleic acid synthesis (10). This analysis also indicated that oncogenic *KRAS* mutations enhance glucose consumption in PDAC through the increase in transcription of the glucose transporter 1 (GLUT1, also known as solute carrier family 2 member 1 [SLC2A1]) the enzymes hexokinase 1 and hexokinase 2 (HK1 and HK2), and lactate dehydrogenase A (LDHA) (**Figure 2**). Thus, *KRAS* contributes to the unregulated growth of pancreatic cancer cells, and directly targeting metabolic pathways as a therapeutic target is a major challenge (93).

We previously showed that mutant *KRAS*, which is a major driver gene in PDAC cells, acts in a eukaryotic translation initiation factor 4A (eIF4A)-dependent manner to promote the translation of ARF6 mRNA, which is a member of the ARF family of GTPases with a quadruplex structure in the 5′-untranslated region, and upregulates ARF6 protein expression (94). Recently, it was also reported that silencing of ARF6 inhibits the Warburg effect, which is associated with aerobic glycolytic processes, in *KRAS*-mutated PDAC cells (95). The oncogene *c-Myc* is a transcription factor that regulates aerobic glycolysis through the upregulation of many key glycolytic genes, such as *GLUT1, HK2*, and *LDHA* (96, 97), and is associated with the transcriptional activation of ARF6. ARF6 has also been shown to be associated with the regulation of the expression of *GLUT1, LDHA*, and *HK2*, as well as *c-Myc* (95). Thus, it is possible that ARF6 is involved in the regulation of aerobic glycolysis *via* the regulation of *c-Myc* in PDACs. Interestingly, in several cancers, including PDAC, the upregulation of GLUT1 in cancer cells correlates with the low infiltration rate of cytotoxic CD8^+^ T cells (98–100). This suggests that tumor cells successfully compete for glucose, suppressing antitumor immunity while simultaneously maintaining high metabolic and proliferative rates (101, 102). Importantly, this also indicates that antitumor immune cells are unable to obtain sufficient energy, thus impairing their function.

As solid tumors progress, large areas of the tumor often become deprived of oxygen, which interferes with ability of the immune system to combat the tumor (103). PDAC is characterized by a very hypoxic TME, and it has been noted that the high malignancy and poor curative efficacy of PDAC are mostly due to the hypoxic TME (103, 104). PDAC also shows increased accumulation of stromal tissue, i.e., desmoplasia, which may collapse blood vessels, and subsequently impede perfusion and promote maintenance of the hypoxic TME. Hypoxia and desmoplasia induce the expression of HIF-1α and its stabilization (105). HIF-1α is a key regulator of cellular responses to changes in oxygen concentration, and supports tumor cell adaptation to hypoxia in an oxygen-deprived TME.

Under hypoxic conditions (usually below 3% to 5% O_2_), the HIF-1α subunit stabilizes and forms a dimer with the β-subunit aryl hydrocarbon receptor nuclear translocator (ARNT), which translocates to the nucleus to promote O_2_-regulated gene expression. HIF-1α is considered to play a crucial role in of metabolic reprogramming (106). Several studies have confirmed that HIF-1α meets the metabolic needs of pancreatic cancer cells by increasing the expression of glycolysis-associated enzymes and the production of lactate (107–110). Indeed, the stabilization of HIF-1α has been reported to induce GLUT1 expression in a HIF-1α -dependent manner, increasing cellular glucose uptake and supporting aerobic glycolysis in cancer cells (111, 112). HIF-1α is also known to enhance the expression of LDHA (113, 114) and monocarboxylate transporter 4 (MCT4; encoded by *SLC16A3*) (115). LDHA reduces the dependence on oxygen-dependent mitochondrial oxidative phosphorylation (OXPHOS) by converting pyruvate to lactate, and the cell preferentially uses oxygen-independent glycolytic pathways to maintain sufficient ATP production to meet bioenergetic requirements, whereas MCT4 removes lactate from the cell by transporting it out of cells. Thus, HIF-1α drives the conversion from oxidative to glycolytic metabolism during hypoxia, which is not only beneficial for bioenergetic homeostasis, but may also promote tumor survival and growth.

Interestingly, it has been shown that the stabilization of HIF-1α by di-methyl-oxaloylglycine treatment markedly increases the level of ARF6 mRNA (116), and ARF6 activity is significantly promoted under hypoxia (117). As mentioned previously, ARF6 is also associated with the enhanced expression of genes involved in glycolytic metabolism in malignant pancreatic cancer with *KRAS* mutations, so hypoxia may potently promote glycolytic metabolism through the induction of HIF-1α and ARF6, thereby regulating the adaptive responses to a hypoxic environment.

In addition to glucose deprivation *via* tumor cells in the TME, higher rates of aerobic glycolysis in tumor cells may promote the production of lactic acid, which in turn increases the acidity of the TME. Excess lactate produced in tumor cells can also suppress CD8^+^ T and NK cell activation, and enhance the function of immunosuppressive cells, such as myeloid subsets, and M2-polarized macrophages to an immunosuppressive phenotype (118, 119). This makes it difficult for immune cells to survive, but tumor cells can often adapt, survive, and multiply despite these harsh conditions. Tumor cells can respond to extracellular acidic pH conditions and regulate cellular acid homeostasis by altering the expression of proteins associated with pH regulation, such as monocarboxylate transporters and carbonic anhydrase (CA) (120). In *in vitro* models of melanoma, exposure to lactic acidosis has been shown to induce the EMT phenotype (121). In pancreatic cancer cells, lactate enhances the expression of IL-8 and contributes to EMT and metastasis (122–124), and tumor cells can use lactate as an alternative energy fuel to promote their proliferation (125). Indeed, high levels of lactate in PDAC have been shown to correlate with poor patient prognosis (126). Therefore, it is strongly suggested that the acidic environment in tumor tissue is involved in the acquisition of mesenchymal traits, and the augmentation of an immunosuppressive PDAC TME.

### Lipid metabolism

Lipids are major components of biological molecules, and play important roles in various processes. Lipids are composed of thousands of different molecules, including phospholipids, sphingolipids, fatty acids, cholesterol, cholesteryl esters, and triglycerides. Such lipids are implicated in a variety of cellular processes, and are important components of biological membranes (127–132). Lipid uptake, accumulation, and lipogenesis are increased in various cancers, including pancreatic cancer, and provide energy for rapid tumor growth. In the early step of *de novo* lipid synthesis, ATP-citrate lyase (ACLY) catalyzes the conversion of citrate to acetyl-CoA, which is then converted to malonyl-CoA by acetyl-CoA carboxylase. The acyl groups of malonyl-CoA and acetyl-CoA bind to the acyl-carrier protein domain of fatty acid synthase (FASN) in an NADPH-dependent way to produce long-chain saturated fatty acid (133) (**Figure 2**).

Expression levels of lipogenic enzymes, including ACLY, are known to be often upregulated in PDAC (134, 135). Inhibition of ACLY activity suppresses PDAC cell growth in xenograft tumor models (136). Furthermore, PDAC patients, highly expressing FASN, have been shown to have shorter overall survival than those expressing low levels of FASN (137). Overexpression of the *FASN* gene may be correlated with resistance to radiotherapy and gemcitabine in pancreatic cancer patients (138), and inhibition of FASN results in high cytotoxicity of this drug. As higher lipogenic activity has been shown in PDAC cells compared with normal cells, genetic and pharmacological inhibition of FASN and other lipogenic enzymes appears to be a promising therapeutic strategy.

The mevalonate pathway (MVP) is essential for cellular lipid metabolism, including cholesterol biosynthesis and the post-translational prenylation of proteins (139). The rate-limiting enzyme in this pathway, 3-hydroxyl-3-methylglutaryl-CoA (HMG-CoA) reductase, has been considered as a prominent target for MVP inhibition, and is increased in a KRAS-driven PDAC mouse model (125, 140). Statins, which are reductase inhibitors, are used for the treatment of hypercholesterolemia (141). The anticancer effects of statins have also been analyzed *in vitro* in various cancer cell lines. Several studies have reported that simvastatin inhibits cancer cell proliferation by promoting apoptosis and reducing cell cycle progression *via* several kinds of signaling pathways, including mitochondrial apoptotic signaling pathways and the Rho signaling pathway involved in cell cycle arrest (142, 143). In addition, lipophilic statins (lovastatin, simvastatin, etc.) have been shown to be potent vaccine adjuvants *via* modulation of post-translational protein prenylation. Mechanistically, statins inhibit geranylgeranylation of the small GTPase Rab5, such as in antigen-presenting cells, causing inhibition of endosome maturation, sustained antigen retention, reinforced antigen presentation, and activation of T cells (144). Therefore, the MVP pathway is a potential target for cancer immunotherapy.

We have previously shown characteristic features that predict responders of MVP-based cancer treatment. We found that the Arf-GTPase ARF6, and its downstream effector AMAP1 (also called ASAP1/DDEF1), are often overexpressed in various types of cancer, including PDAC, and closely associated with poor patient survival (145–149). Interestingly, we found that the MVP is crucial for ARF6 activation in breast cancer cells. In this process, the MVP is essential for geranylgeranylation of RAB11b, which promotes intracellular trafficking of ARF6 to the plasma membrane where it is activated by RTKs. Furthermore, consistent with reports that gain-of-function mutants of p53 activate the MVP, it is clear that mutant p53 is essential for ARF6 activation (148, 150). Our *in vitro* experiments showed that the presence of statins improved the sensitivities of breast cancer cells to various drugs. In contrast, inhibition of MVP is ineffective when cancer cells do not overexpress components of the ARF6-based pathway. We have also shown that statins inhibit not only ARF6 activity and invasive potential but also recycling of the immune checkpoint molecule PD-L1 to the plasma membrane in pancreatic cancer cells (94). The chemopreventive effects of statins have been shown in pancreatic cancer cell lines (151–153) and pancreatic cancer model mice (154). Thus, the MVP may be crucial for promoting cancer cell invasion, metastasis, drug resistance, and PD-L1 recycling through the overexpressed ARF6 pathway activated by RTKs.

### Glutamine metabolism

Glutamine addiction is common in various cancers, including PDAC (155–160). Glutamine may be a mitochondrial substrate for synthesis of macromolecules in cancer cells by supplying carbon to fuel the TCA cycle, and is a major nitrogen donor for the production of nucleotides and nonessential amino acids (155). In mitochondria, glutamine has essential roles in the synthesis of energy in the form of ATP through the TCA cycle and the OXPHOS process. Mitochondrial metabolism has been demonstrated to be important for tumor growth in several types of cancer, including PDAC (161, 162). Glutamine is the most abundant nonessential amino acid in the blood and plays various roles in cell metabolism (158, 163). Glutamine is first catalyzed to glutamate by the enzyme glutaminase. Glutamate is then converted to α-ketoglutarate through a deamination reaction catalyzed by glutamate dehydrogenase in the mitochondria. Subsequently, α-ketoglutarate enters the TCA cycle to supply metabolic intermediates, such as citrate and malate, producing NADH and FADH2 to generate ATP. Malate is converted to pyruvate leading to NADPH production, and then pyruvate is in turn transformed to lactate. Glutamine can also produce substantial amounts of the cofactor NADPH by glutaminolysis, in which malate is converted to pyruvate by malic enzyme. Glutamine-derived α-ketoglutarate is reductively carboxylated by mitochondrial isocitrate dehydrogenase 2 (IDH2) to isocitrate, which can then be isomerized to citrate. Citrate produced in the mitochondrial matrix is transported to the cytoplasm and then converted to isocitrate by aconitase in a reversible reaction. Cytosolic isocitrate is metabolized to α-ketoglutarate through cytosolic isoform of IDH1, which can also produce NADPH, which may be used for lipid synthesis. PDAC cells maintain cellular redox homeostasis, which is necessary for cell growth, by metabolizing glutamine in response to NADPH (157).

Circulating glutamine can be taken up *via* transporters, such as alanine-serine-cysteine transporter 2 (ASCT2, also known as SLC1A5), and can be exported or imported *via* large neutral amino acid transporter 1 (LAT1, also known as SLC7A5), in exchange for branched-chain amino acids (BCAAs; leucine, isoleucine, and valine). BCAAs are broken down by branched-chain amino acid transaminase 1 (BCAT1) on the cytosolic side and BCAT2 on the mitochondrial side to produce branched-chain α-keto acid and glutamate (**Figure 2**). Early-stage pancreatic cancer driven by mutant *KRAS* has been shown to increase plasma BCAA levels (164). BCAT2, but not BCAT1, has been shown to be highly expressed in PanIN and PDAC ductal cells. Thus, it has been noted that the BCAA-BCAT2 axis driven by KRAS is important for PDAC development (165). In addition, some amino acid transporters (ASCT2 and LAT1) are overexpressed in pancreatic cancer (166), and associated with poor prognosis. PDAC cells are known to be highly dependent upon glutamine for tumor growth (157, 167). However, whereas the treatment of BPTES, a glutaminase inhibitor to target the glutamine metabolism, significantly inhibited PDAC proliferation, it did not affect PDAC cell death. Glutamine deprivation has been reported to activate macropinocytosis-associated autophagy and maintain proper intracellular glutamine levels by regulating glutamine metabolism. Furthermore, both glutamine deprivation and autophagy inhibition have been shown to robustly activate apoptotic cell death (168). Glutamine plays various roles in PDAC metabolic processes, suggesting that therapeutic strategies targeting the acquisition and utilization of this amino acid may be promising. However, glutamine deprivation was shown to promote the EMT signature *in vitro* and *in vivo* through an increase in the EMT master regulator Slug *via* ERK signaling and ATF4 activation (169). Thus, evaluating the effects of the simultaneous inhibition of distinct aspects of glutamine metabolism, such as the induction of autophagy and EMT on PDAC growth and metastasis may lead to new therapeutic approaches.

Recently, comprehensive analysis of metabolic enzymes by large-scale targeted proteomics demonstrated an enhanced metabolic system in malignant cancers to utilize glutamine-derived nitrogen for DNA synthesis (a shift in glutamine nitrogen metabolism) (170). In malignant cancer cells, the expression level of the metabolic enzyme phosphoribosyl pyrophosphate amidotransferase (PPAT), which transfers the nitrogen from glutamine to nucleic acid precursors, was markedly increased, whereas the metabolic enzyme responsible for glutaminolysis, namely, glutaminase (GLS) was decreased, indicating a shift toward nucleotide biosynthesis. In addition, meta-analyses of human cancers have shown that PPAT is most strongly associated with malignancy among the metabolic enzymes, particularly prominent in neuroendocrine cancers, including small cell lung cancer (SCLC) (170). Interestingly, the hazard ratio for PPAT is high in pancreatic cancer, whereas GLS expression does not significantly correlate with cancer prognosis. In PDAC mouse models, GLS inhibition does not demonstrate any anti-tumor effects *in vivo*, indicating an adaptive metabolic network that sustains proliferation (171). In cancers in which glutamine supply from the circulation is limited, such as PDAC, glutamine synthesis mediated by glutamate ammonia ligase, an enzyme involved in *de novo* glutamine synthesis, and the associated nitrogen assimilation and transfer to nitrogen-containing macromolecules, such as nucleotides, has been shown to be important (172). Thus, shifts in glutamine nitrogen metabolism that promote nucleotide biosynthesis *via* the increased expression of PPAT while suppressing the GLS response, as demonstrated in SCLC, are important in cancer malignancy, and may be a potential therapeutic target for pancreatic cancer in a glutamine-limited environment.

### Autophagy/micropinocytosis

PDACs also rely upon metabolic pathways, such as autophagy and macropinocytosis, to survive and maintain metabolic homeostasis in harsh environments, such as those with low nutrient levels, hypoxia, desmoplasia, and high interstitial pressure. Autophagy is an indispensable intracellular pathway that provides intracellular energy by degrading unnecessary organelles and macromolecules in response to stimuli, such as starvation and accumulation of unfolded proteins (173). The molecular mechanism of autophagy is strictly regulated by more than 30 autophagy-related (ATG) proteins that are responsible for the dynamic autophagy pathways, and can be divided into the following series of steps: phagophore (isolation membrane) growth, closed double-membrane vesicle (autophagosome) formation, autophagosome-lysosome fusion, degradation within the lysosome, and recycling of the degradation products.

One of the characteristic features of PDAC is known to be increased autophagy. This is because owing to the tumor microenvironment of PDAC, in which the low vascular density results in severe hypoxia and limited nutrient utilization (61, 174), PDAC cells must rewire their metabolism to sustain proliferation. Indeed, the inhibition of autophagy by the genetic or pharmacological inhibitor chloroquine (an inhibitor of lysosomal acidification) resulted in mitochondrial metabolic abnormalities leading to decreased OXPHOS, reduced proliferation *in vitro*, and inhibited tumor growth *in vivo* (61). Furthermore, the significance of autophagy in PDAC tumorigenesis was confirmed by crossing a conditional knockout mouse of the autophagy essential gene *Atg5* with a PDAC mouse model (175, 176). This autophagy inhibition in mouse studies may exert anti-tumor effects by cooperating with the TME (177). Indeed, the crosstalk between stromal cells and tumor cells in PDAC is important, indicating that autophagy is required for stromal cells to secrete alanine, which is then taken up by tumor cells to support their growth (178). In a study using a PDAC mouse model expressing a tetracycline-inducible dominant-negative ATG4B protein which can reversibly and acutely inhibit autophagy in fully formed tumors, the inhibition of autophagy was shown to suppress tumor growth *via* intrinsic as well as extrinsic factors in tumor cells (61). This study also showed that the effect of inhibiting autophagy in the tumor itself on tumor regression was partially mediated by macrophages, indicating that induction of the immune system *via* autophagy inhibition is also important for the anti-tumor effects. This may mean that there is autophagy-dependent metabolic crosstalk between tumor cells and the stroma, and hence autophagy is necessary to support the metabolism, tumorigenesis, and survival under harsh conditions of tumors.

PDAC does not respond well to ICIs, such as anti-PD1 and anti-CTL4A antibodies, and typically has a highly immunosuppressive TME that is characterized by marked infiltration of myeloid-derived suppressor cells (MDSCs) and lack of active cytotoxic CD8^+^ T cells (179–182). Resistance to ICI therapy is known to be associated with major histocompatibility complex class I (MHC-I), which is essential for endogenous antigen presentation by cancer cells (183–185). PDAC cells have been shown to have reduced expression of MHC-I molecules on the cell surface, and instead localize predominantly to autophagosomes and lysosomes (186, 187). Indeed, it has been demonstrated in human and mouse PDAC that MHC-I is degraded by an autophagy-dependent mechanism to induce immune evasion (188). In addition, autophagy inhibition increased the surface levels of MHC-I, leading to the promotion of antigen presentation, enhanced anti-tumor activity of T-cell responses, and suppression of tumor growth in orthotopically transplanted syngeneic mice. Systemic autophagy inhibition by chloroquine, as well as the tumor-specific inhibition of autophagy, in combination with ICIs, showed synergistic effects. These findings provide a molecular mechanism by which autophagy promotes immune evasion, and provide a rationale for further research toward the development of new therapies targeting the autophagy-lysosome system in PDAC.

When glucose is deprived in PDAC cells, large amounts of reactive oxygen species are produced to activate autophagy, and provide the nutrients necessary for growth (189). On the other hand, glutamine starvation increases the degree of macropinocytosis in PDAC cells, and hence glutamine is important for regulating the degree of macropinocytosis in PDAC cells (190). Macropinocytosis is a process involving membrane ruffles, which are used to internalize extracellular materials, such as soluble molecules, nutrients, and antigens. After the nonspecific uptake of extracellular fluids by endocytic processes, the formation of vesicular structures, named macropinosomes, which contain the internalized proteins fuse with lysosomes, resulting in proteolytic degradation. The free amino acids produced by this process support the metabolic requirements of tumor cells (191). Thus, macropinocytosis is a nonselective endocytotic program capable of taking up content from extracellular fluid in a nutrient recycling and scavenging pathway that has been recognized as a key mechanism supporting pancreatic cancer growth (192).

PDAC cells expressing oncogenic *KRAS* mutation exhibited high enhancements of basal macropinocytosis consuming extracellular proteins for rapid tumor proliferation, which is closely linked to autophagy (174, 193–198). It has been shown that autophagy is required for the micropinocytosis-mediated degradation of extracellular proteins, and autophagy plays an important role in the breakdown of macromolecules internalized by macropinocytosis, to provide amino acids, particularly glutamine, in PDAC cells (168). The dynamic balance between glutamine metabolism and macropinocytosis-associated autophagy may ensure PDAC cell growth. Although these studies suggest that macropinocytosis is a potential therapeutic target for PDAC, understanding how macropinocytosis and autophagy cooperate is crucial for establishing treatments for PDAC.

ARF6 has been shown to regulate autophagy and colocalize with proteins mediating the initiation of autophagosome formation, i.e., the formation of pre-autophagosomal structures and phagophores (199, 200). Mechanistically, activation of the lipid-modifying enzyme PIP5K by ARF6 may contribute to autophagy, as PIP2 produced by PIP5K affects membrane trafficking for phagosome formation, by regulating plasma membrane endocytosis. Interestingly, ARF6 has been shown to be required for macropinocytosis in HT180 cells, a human fibrosarcoma cell line (201). In PDAC expressing high levels of ARF6, ARF6 may be a potential target for autophagy and micropinocytosis, and combination therapy, such as ICIs, may lead to a new treatment for PDAC. We also demonstrated that combination therapy with the eIF4A inhibitor silvestrol, which inhibits ARF6 protein production, and anti-PD-1 antibodies improves the efficacy of anti-PD-1 therapy in PDAC (202). However, it remains unclear whether ARF6 inhibition actually affects therapeutic efficacy by inhibiting autophagy and macropinocytosis.

### Other types of metabolism

Amino acid availability in the TME, particularly arginine and tryptophan, is an important determinant of antitumor immunity. Increased arginine levels play an important role in T-cell activation by inducing metabolic changes, including a shift from glycolysis to OXPHOS, and the promotion of memory T-cell differentiation (203). Indoleamine 2,3-dioxygenase (IDO), which catalyzes the conversion of tryptophan to kynurenine, is often overexpressed in PDAC (204). Tryptophan depletion and kynurenine production in TME promote the establishment of a suppressive immune environment, and attenuate anti-tumor T-cell responses (205).

Extracellular ATP levels may be rapidly and robustly increased by hypoxia (206, 207). ATP, which has immunostimulatory properties on its own, may be ultimately converted to the nucleoside adenosine through stepwise process. Canonically, ATP is first catalyzed to AMP *via* the ectonucleotidase CD39. AMP is then dephosphorylated by CD73 and degraded into adenosine. Adenosine can then act on purinergic receptors, such as A1, A2a, A2b, and A3 (208), and regulates various aspects of physiology and pathophysiology (209, 210). A2a receptors and A2b receptors are primarily responsible for the downstream signaling of immunosuppression associated with intracellular cAMP accumulation (211). In PDAC, high expression of CD73 was demonstrated to be associated with an immunosuppressive TME and poor survival, as well as decreased CD4^+^, CD8^+^, and CD21^+^ TILs (212). Therefore, CD73 may also play a significant role in regulation of the immunosuppressive microenvironment of PDACs and promote their tumor progression.

## Immunosuppressive TME in PDACs

The emergence of cancer immunotherapy, particularly ICIs, has offered hope to many patients with tumors that are not curable by conventional therapies. However, PDAC is known to be less sensitive to ICIs than other solid tumors, such as melanoma and lung adenocarcinoma. On the other hand, in PDAC patients, neoantigen quality has been shown to be associated with overall survival, suggesting that PDAC is associated with acquired immunity (213). In particular, the preclinical success of ICI therapy in PDAC patients with microsatellite instability (MSI high) and mismatch repair defects, as well as the therapeutic potential of autologous T-cell-based therapy in PDAC patients, holds promise for adaptive immune-based treatment strategies for PDAC (214, 215). At present, there is an ongoing study testing the effects of ICIs in patients with MSI-high PDACs (NCT02628067), which may provide insights into the subset of patients who respond to immunotherapy and the underlying mechanisms related to efficacy and resistance for ICIs. Overall, clinical results have been disappointing, but in some cases, correlative immunophenotypic studies have demonstrated that these therapies elicit adaptive T-cell responses. This suggests that immunosurveillance is operating in PDAC, however, a rational approach to countering its highly heterogenous and plastic immune evasiveness is needed.

### TME of PDACs

**Figure 3:**
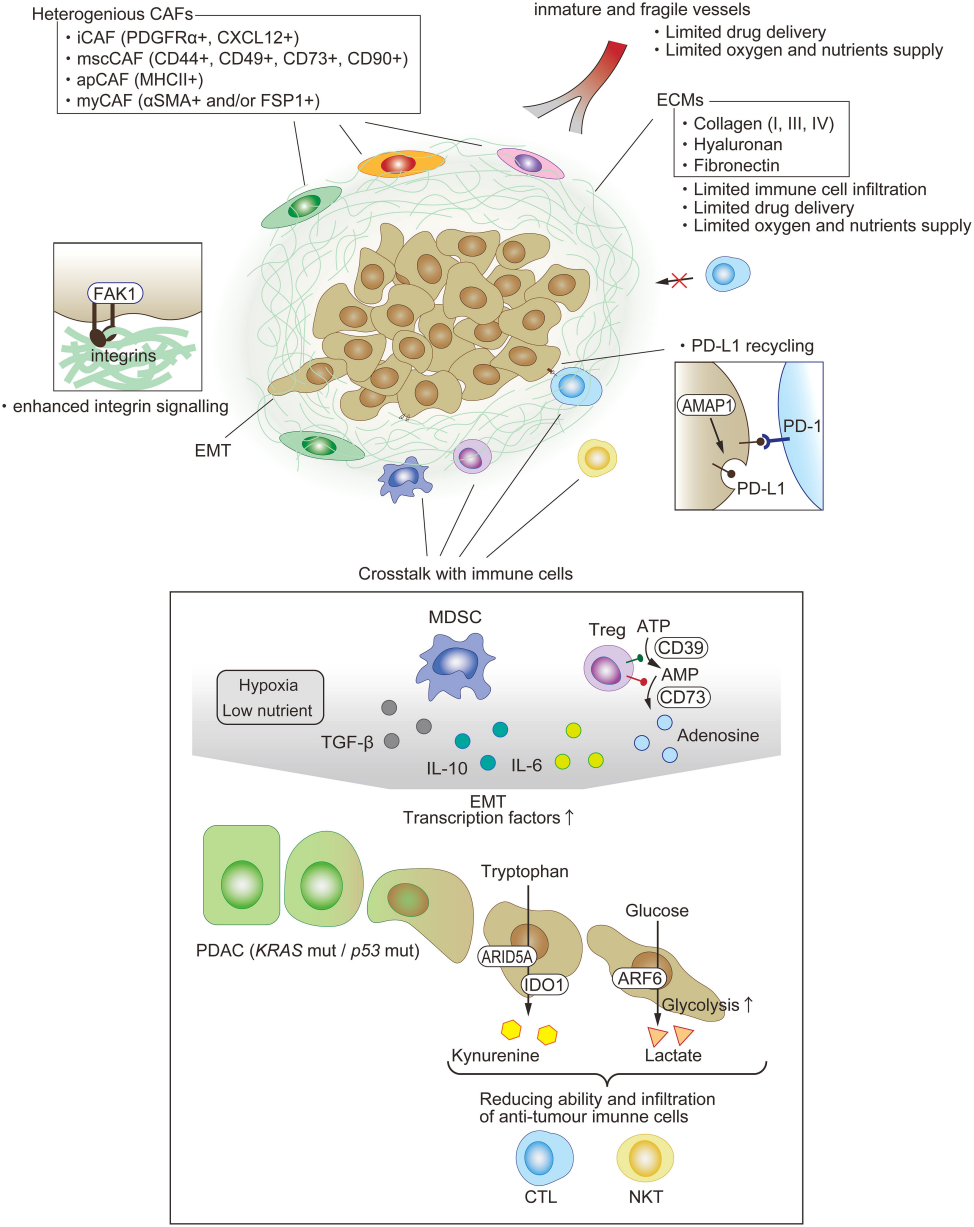
Immunosuppressive TME in PDAC. One of the features of PDAC is dense fibrosis, which limits immune-cell filtration, drug delivery, and oxygen supply. A variety of cells exist and compose the immunosuppressive milieu. Each type of fibroblast appears to play a key pro-tumor or anti-tumor role. The EMT of PDAC is among the factors enhancing anti-tumor immunity through not just cell intrinsic functions, such as PD-L1 recycling, but also crosstalk with other immune cells.

Pancreatic cancer is known to have an immunologically cold microenvironment. Overall, immunosuppressive TME in PDAC is often associated with the presence of a tumor-promoting immune cell population (216). Analysis of PDAC mouse models has shown that the expression of oncogenic KRAS itself leads to robust inflammation, and initiates the cycle of inflammation associated with carcinogenesis (11, 179, 217, 218). Furthermore, whereas the expression of KRAS mutant during embryogenesis is sufficient to promote the onset of PDAC proliferation, chronic inflammation is required for malignant transformation in adult PDAC mouse models, indicating that oncogenic mutations alone cannot induce PDAC malignancy (97, 219, 220). Therefore, the inflammatory environment and oncogenic mutations work in concert to promote tumor progression. Thus, inflammation caused by cytokines and chemokines released from PDAC cells that have acquired mesenchymal traits is often associated with the infiltration of innate immune cells that facilitate an immunologically tolerant environment rather than an antitumor immune response (221). A low level of T-cell infiltration correlates with mortality in PDAC (222). Biochemical (production of chemokines and other factors in TME) and physical (deposition of the ECM) barriers in the stroma surrounding the TME inhibit T-cell infiltration (**Figure 3**).

### Fibrosis

Although there are multiple factors that cause ICI treatment resistance, one of the main possible contributors is a dense fibrous stroma (desmoplasia) occupying 80% to 90% of the tumor mass in PDAC (223, 224). Desmoplasia is caused by the proliferation of α-smooth muscle actin-positive fibroblasts or activated pancreatic stellate cells, and work as a physical barrier against drug and immune cells. The trigger that causes these cells to proliferate is still unknown, but the communication among tumor cells and these cells have been identified. Two main components constitute desmoplasia: cells including fibroblasts and infiltrating immune cells, and noncellular proteins, such as collagen types I, III, and IV, fibronectin, and hyaluronan. A comprehensive review of the pancreatic cancer stroma has been published recently (15).

### Heterogeneous fibroblasts

Fibroblasts exist in every solid organ, to maintain their morphology and function by depositing ECM proteins and secreting soluble factors (225). For instance, TGF-β secreted from fibroblasts is used by epithelial cells to cure skin injuries. Histological similarities, such as mesenchymal morphology, are maintained among fibroblasts in various organs, but their genomic landscapes differ depending on the organ in which they are located (226). Many studies have demonstrated that some fibroblasts contribute to tumor initiation, progression, and metastasis, and they are known as CAFs (227). Pancreatic cancer has a dense fibrotic architecture, and therefore, it will be useful to clarify the biology of CAFs in PDAC. Recent studies have demonstrated that the functional roles of CAFs in PDAC TME are more complicated than their expected simple tumor-promoting role (15).

Three-dimensional *in vitro* coculture of pancreatic stellate cells (PSCs) and KPC mouse-derived PDAC organoids induced two kinds of CAFs (228). Cocultured directly, PSCs turned into myofibroblastic CAFs (myCAFs) with highly upregulated α-SMA expression and myofibroblastic gene profiles (228). Although CAFs are thought to literally be ‘associated’ with tumors, myCAFs have anti-tumor activity (229), which requires further investigation for elucidation of the mechanism. On the other hand, indirect coculture transforms PSCs into inflammatory CAFs that display inflammatory cytokines, such as IL-6. It is thought that CAF-derived IL-6 contributes to immune evasion (228).

scRNAseq by several investigators supported the existence of these two populations, and moreover, identified two other groups, namely, mesenchymal stem cell CAFs (mscCAFs) and antigen-presenting CAFs (apCAFs) (230, 231). MscCAFs are characterized by the expression of previously identified mesenchymal stem cell markers (CD44, CD49a, CD73, and CD90), and originate from bone marrow (232). They preferentially express GM-CSF, thus promoting macrophage polarization towards an immunosuppressive phenotype that results in the inhibition of CTL activity (179, 233, 234). Coexpression of MHC class II, including CD74 and podoplanin, a pan-CAF marker, is a signature of apCAFs (230). It has recently been reported that apCAFs are derived from mesothelial cells and can induce the transformation of naïve CD4^+^ T cells into regulatory T cells by their direct ligation with a specific antigen (235). This group also demonstrated that targeting mesothelin expressed in the mesothelium may be an effective treatment owing to the inhibition of apCAF formation and regulatory T (Treg) cell induction (235).

### T cells

T-cell exclusion in tumors is primarily mediated by CAFs that express fibroblast activation protein, and secrete the chemokine CXCL12 (236). Additionally, activation of integrin-binding protein and non-receptor tyrosine kinase focal adhesion kinase (FAK) is associated with increased collagen I deposition and immunosuppression (237, 238). Highly phosphorylated FAK levels in pancreatic cancer patients were associated with decreased tumor-infiltrating CD8^+^ T cells and reduced survivability (239). In addition, the expression of FAK in patients with PDAC is associated with decreased tumor cellularity and survival (239).

Acquiring a terminally differentiated T-cell state can diversely impact disease outcome, either countering tumor proliferation through antigen-limited tumor-killing immune responses, or promoting cancer progression by actively inducing immunosuppression (216, 240, 241). In particular, CD8^+^ cytotoxic T lymphocytes and polarized CD4^+^ T cells known as T helper type 1 (Th1) cells exert protective effects against tumors in PDAC mouse models, and have been shown to be associated with prolonged survival of human PDAC patients (242). Conversely, CD8^+^ T-cell deficiency, low amounts of neoantigens, and CD4^+^ Th2 and Treg cells are associated with tumor-permissive anergy (242–245). Cytokines produced by Th2, particularly IL-4 and IL-13, can not only reduce anti-tumor immune responses, but also can directly accelerate tumor growth induced by KRAS transformed cells (246). PDAC tumors are also accompanied with abundant lymphocyte infiltrates that are typically associated with the gastrointestinal mucosa (247). Th17 cells comprise approximately 5% of the CD4^+^ T cells in PDACs. The role of Th17 cells in the TME is also context-dependent. In PDAC, IL-17 secretion from γδ T cells and Th17 cells may enhance antitumor immune responses (248). However, early stages in PDAC carcinogenesis, IL-17 has a direct mitogenic effect on *KRAS* mutation-induced PanIN cells expressing IL-17R (218). Whereas the effects of distinct T-cell subsets depend on the underlying immune context of the tumor due to various physiological conditions and environments, and may be altered during the tumor progression of PDAC, the regulation of differentiation and function of T cells in PDAC TMEs play crucial roles in tumor immunity.

### B cells and myeloid cells

To date, it has become clear that distinct cell populations derived from lymphocyte and myeloid cells can act in a pro- or anti-tumor manner, depending on the situation. B-cell subsets have become apparent as key immunomodulators in PDAC (249–252). Furthermore, suppressive myeloid cell programming is a major cause of tolerogenic T-cell programming in PDAC. Macrophages are thought to serve a major function in the induction of immunosuppression in PDAC; IL-10^+^Arg1^+^MHCII^lo^ tumor-associated macrophages (TAMs) are predominant in the PDAC TME, and are effective in promoting Th2 cell differentiation, but ineffective in inducing CD8^+^ T-cell immunity (253–257). Similarly, immature MDSCs, collectively referred to as bone marrow-derived Gr1^hi^CD11b^+^ granulocyte lineage MDSCs, are characterized by a short half-life and strong suppressive effects in the TME (258). Although endogenous normal dendritic cells (DC) in the TME can produce anti-tumor T cells, the number of DCs in PDAC is low and probably insufficient to sustain robust adaptive immune responses. Furthermore, tumor-derived colony-stimulating factor 3 is found to inhibit the development process of DC in the bone marrow (259). Certain DC subsets have been understood as activators of immune evasiveness in PDAC. CD11b^+^CD103^−^ DCs with high expression of IL-23 and TGF-β are predominant in PDAC, drive the differentiation of FoxP3^−^ tumor-promoting type I^+^ T cells, and promote metastatic spread (260, 261). Moreover, it has been shown that Treg cells directly interact with tumor-associated DCs and suppress anti-tumor immunity by downregulation of costimulatory ligands expression that are important for activation of CD8^+^ T-cell (262).

Stimuli that recruit myeloid cells to the TME in PDAC are only partially understood. In mouse models, tumor-derived factors have been presented to accumulate MDSCs in the PDAC TME. In the same way, CCL2 produced by tumor cells and CSF1 produced by tumor-associated fibroblasts contribute to the generation of M2-like macrophages (263, 264), and CXCL1 production by tumor cells has been linked to increased myeloid cell populations and decreased tumor infiltration of cytotoxic CD8^+^ T cells (265). In particular, focusing on the CSF1-CSF1R, CCL2-CCR2, and CXC chemokine-CXCR2 axes to target in the PDAC TME may contribute to pancreatic cancer progression.

### Microbiota

The normal pancreas has long been believed to be a sterile, protected site from bacteria. However, recent studies have shown that the pancreas contains bacteria that invade through the Vater’s vastus. Interestingly, it has been reported that in the inflammatory environment of PDAC, the bacterial content of pancreatic tumor tissue increases by approximately 1,000-fold compared with normal tissue (266–268). Furthermore, the bacterial species found in the tumorigenic pancreas are different from those in the gut, and low microbial diversity in the tumor results in a low survival rate of patients with PDAC, whereas high tumor microbiome diversity is associated with long-term survival (269). Mechanistically, the primary PDAC microbiome has a potent immunosuppressive effect on the inflammatory TME, driving the protumor inflammatory responses of PDACs *via* the activation of Toll-like receptors on bone marrow-monocyte cells, and inducing the expansion of MDSCs and anti-inflammatory M2-polarized macrophages. These innate immune cells with tolerogenic functions enhance the differentiation of immunosuppressive CD4^+^ T-cell populations and inhibit the expansion of cytotoxic CD8^+^ T cell populations (268). Consistently, microbial ablation in mice resulted in increased infiltration of Th1-polarized CD4^+^ and CD8^+^ T cells, decreased accumulation of MDSCs, and a TAM reprogrammed to a tumor-protective M1-like phenotype (268, 270). Potentially, targeting the microbiome by oral antibiotics might reverse myeloid cell-mediated adaptive immunosuppression and promote the efficacy of ICI therapy in PDAC.

## Novel mechanisms bridging mesenchymal malignancy and immune evasiveness *via* rewiring of the metabolic program of PDAC

Immune evasion is an essential characteristic of cancer. Every day, the adult body produces mutant cells owing to genetic mutations *via* various intrinsic and extrinsic factors, and most mutant cells are detected and eliminated by the immune surveillance system. However, in rare cases in which mutant cells acquire traits that enable them to evade the immune surveillance system, the cells evade attack by immune cells and proliferate, eventually manifesting as cancer.

As mentioned above, the major immunosuppressive factors in the TME of PDAC include hypoxia, a low-nutrient environment, expression of immune checkpoint molecules, accumulation of immunosuppressive cell populations, such as Tregs and MDSCs, production of immunosuppressive cytokines, such as TGF-β and IL-10, immunosuppressive metabolic enzymes, such as Ido, arginase, and CD39/CD73, and metabolites, such as lactate and kynurenine. In addition, cancer-associated inflammation induced by IL-6, IL-1, IL-17, IL-22, and IL-23 is not only a driver of carcinogenesis, but is also associated with tumor progression by inducing EMT, whereby epithelial cells acquire malignant mesenchymal properties, such as detachment from other cells, invasion into adjacent tissues, and accelerated metastatic spread to other distant organs (21, 271–275). Thus, it is easy to speculate that the factors involved in the suppression of the immune environment of the TME are diverse and complex in their mechanisms of action, as they are produced not only by cancer cells, but also by the various stroma cells, including several kinds of immune cells and heterogenous fibroblasts in the TME.

On the other hand, international cancer-related consortiums, such as The Cancer Genome Atlas (TCGA) have promoted comprehensive genome-wide gene expression analyses of various cancers, but these efforts have not led to the development of effective diagnosis and treatment methods, particularly in the case of PDAC. There may be various reasons, such as the fact that the collected tissue sections are bulk preparations containing not only cancer cells but also stromal cells. Recently, the transcriptome and proteome have been compared worldwide, and it has been shown that there is a very poor correlation between the mRNA and protein levels of most genes (276–280). This strongly suggests that post-transcriptional mechanisms play an important role in the regulation of gene expression. Here, we present our studies from two different aspects on the molecular mechanisms linking the acquisition of mesenchymal plasticity and immune evasion in PDAC, with a focus on post-transcriptional mechanisms.

### Functional roles of ARF6-AMAP1 axis as a mesenchymal executioner in PDAC

ARFs, a family within the Ras superfamily of small GTPases, are evolutionally the most ancient of the small GTPases. The ARFs are conserved throughout eukaryotes, including in species that branched off early, such as *Giardia lamblia*, in which no members of the Ras family nor heterotrimeric G-proteins are found (281, 282). *Giardia lamblia* is an anaerobic eukaryote parasite of the gut, which is evolutionally inferred to be an amitochondrial-type eukaryote that developed before the creation of mitochondria (283). This implies that eukaryotic cell features, such as nuclei and flagella, predate mitochondrial endosymbiosis, suggesting that ARF family molecules have been deeply involved in the maintenance of life homeostasis under anaerobic conditions during the evolution of eukaryotes. The human ARF family consists of six isoforms, ARF1–6, which are classified into three classes based on sequence homology, as follows: class I (ARF1–3), class II (ARF4–5), and class III (ARF6) (284). Class I and class II Arfs primarily regulate vesicular transport between the Golgi and endoplasmic reticulum (284, 285). Although ARF6, the only class III member, has virtually identical effector-interacting domains as the other ARFs, it is the most divergent of the ARF proteins, and predominantly localizes to the plasma membrane and recycling endosomal compartments, and functions in intracellular events associated with membrane dynamics, including recycling of plasma membrane components (including both endocytosis and recycling-back to the plasma membrane), as well as in actin-cytoskeletal rearrangement at the cell periphery (286–288).

We identified the ARFGAP protein AMAPs as molecules that are induced during macrophage differentiation, bind to the integrin-associated protein, paxillin, and are involved in its intracellular dynamics. Furthermore, we found that AMAPs are ARF6-specific ARFGAP proteins that are commonly involved in enhancement of the cell motility of macrophages and epithelial cells (289–291). In addition, we identified a novel mechanism of action in which AMAP functions as an effector of activated ARF6 through steady-state binding to GTP-bound ARF6 *via* its ARFGAP domain in the presence of Mg^2+^ (290, 291). Consistently, Wittinghofer and colleagues demonstrated that Ca^2+^ spikes stimulate the ARF6-specific GAP activity of AMAPs, but not other members of the ArfGAP family (292).

Subsequently, we identified GEP100 as a guanine-nucleotide exchange factor that activates Arf6 in the acquisition of invasive and metastatic traits of breast cancer cells upon activation of the epidermal growth factor (EGF)receptor pathway (145). We also identified the mechanism of action by which GEP100 activates Arf6 by binding directly to the phosphotyrosine moiety of the activated EGF receptor *via* the Pleckstrin-homology domain. Furthermore, we found that the simultaneous expression of Arf6 and GEP100 in MCF7 human epithelial-like breast cancer cells induced EGF-stimulation-dependent EMT-like changes. Subsequently, pathological analysis demonstrated that GEP100 expression is present in approximately 80% of invasive breast cancers (145). Our present study suggests that Arf6-based signaling pathways play an important role in the acquisition of invasive and metastatic traits *via* EMT induction in cancer cells. In this pathway, AMAP1 binds to different proteins, such as cortactin, and protein kinase D2 to promote cortical actin remodeling and integrin recycling (293, 294). AMAP1 also binds to EPB41L5 (148, 149), which shows increased expression during TGF-β-induced EMT (295). Furthermore, we demonstrated that the EMT-TF ZEB1 is involved in *EPB41L5* gene expression, and that high expression levels of ZEB1 and EPB41L5 in cancer cells are associated with p53 mutations. This study demonstrated that the ARF6 pathway is a signaling pathway responsible for advanced cancer-specific mesenchymal traits associated with mutant p53 (296).

A series of our studies have identified that high protein levels of ARF6, AMAP1, and EPB41L5 were associated with invasiveness of several kinds of solid tumors, including breast cancer, clear cell renal cell carcinoma, lung adenocarcinoma, and PDAC and importantly that these expression levels were statistically correlates with poor prognosis (94, 145, 146, 149).

Notably, *ARF6* and *AMAP1* mRNAs are both rich in G/C content in their 5′-untranslated regions (UTRs) (74% and 88%, respectively) (297). Moreover, *ARF6* mRNA contains a G-quadruplex structure at the 5′-UTR (94), indicating that efficient translation is dependent upon the RNA helicase eIF4A, a member of Cap-dependent translation initiation factors (298, 299). On the other hand, the 5′-UTR of *AMAP1* mRNA contains a 5′-terminal oligopyrimidine-like sequence, indicating the mTOR complex 1 kinase-dependent translation control (300, 301). We found that the eIF4A inhibitor silvestrol suppresses protein levels of ARF6 in *KRAS* mutant cells, but only moderately in KRAS intact cells (202), and the mTOR inhibitors rapamycin and Torin1 suppress AMAP1 expression in *KRAS* mutant cells (94). Mechanistically, oncogenic *KRAS* mutations are the major cause of the aberrant overexpression of ARF6 and AMAP1, in which KRAS signaling enhances eIF4A-dependent *ARF6* mRNA translation and eIF4E-dependent *AMAP1* mRNA translation. In addition, gain of function mutations of *TP53* promoted the activation of ARF6 by PDGF *via* MVP-mediated geranylgeranyl lipid modification of Rab11b in PDAC cells (94, 148). Moreover, we revealed that the ARF6-AMAP1 pathway is closely associated with immune evasion in a KPC mouse model. Thus, the cooperation between eIF4A/4E-dependent mRNA translation and MVP has been identified as a link in which representative pancreatic driver mutations empower an ARF6-based pathway, activated by external ligands, to promote tumor cell motility, PD-L1 dynamics, and immune evasion. A recent clinical study by another group confirmed the importance of ARF6 in this context (302). We hence propose that targeting eIF4A, or eIF4E, as well as mutant KRAS, provides novel methods to improve the efficacy of anti-PD-1 therapy, in which ARF6 and AMAP1 overexpression may act as biomarkers to identify patients with drug-resistant disease in PDAC. Additionally, the ARF6-AMAP1 pathway was also found to be involved in acidosis and fibrosis of the TME, both of which are well known to be barriers against immune attack to cancer cells (81, 303), indicating that the ARF6-AMAP1 pathway may also be a valuable target in modifying the TME from pro-tumor, which makes PDAC resistant to treatment, towards an anti-tumor state. Taken together, given the importance of the ARF6-AMAP1 pathway in the pathophysiology of PDAC, its clinical application as a therapeutic target may broaden options for the treatment of PDAC (**Figures 2**, **3**).

### Arid5a acts as a dual regulator in malignant PDAC to generate an immunosuppressive TME

Arid5a was identified as an RNA-binding protein that binds directly to the 3′-UTR of *Il6* and stabilizes *Il6* mRNA (304). Recent studies have demonstrated that Arid5a plays an important role in innate and adaptive immune responses (305). In macrophages and embryonic fibroblasts, stimulation by LPS, IL-1, and IL-6 induces Arid5a expression (304, 305). Importantly, in untreated rheumatoid arthritis (RA) patients, expression of Arid5a in CD4^+^ T cells is increased, whereas treatment with the anti-IL-6 receptor antibody tocilizumab is associated with decreased Arid5a expression (306), indicating that the IL-6-ARID5a axis may be involved in RA pathogenesis. Consistently, Arid5a has been shown to be involved in several immune-associated pathologies. For example, Arid5a deficiency reduces IL-6 production under LPS-induced endotoxemia. Furthermore, in an experimental autoimmune encephalomyelitis (EAE) model, Arid5a deficiency significantly suppresses Th17 cell differentiation and lowers IL-6 serum levels, resulting in the reduced development of EAE (304). In addition, Arid5a regulates the stability of mRNAs for other genes involved in immune regulation, such as *Stat3*, *Tbx21*, *Ox40*, and *Il17* (307–310). In addition, IL-6 increases its own mRNA stability by increasing Arid5a levels *via* a positive feedback loop (311). Consistently, Arid5a-deficient mice show impaired LPS-stimulated *Il6* and *Ifnγ* expression, and are resistant to lethal endotoxic shock (304, 308). Thus, Arid5a-mediated upregulation of these factors may be involved in the enhancement of Th1 and Th17 cell polarity and function in acute inflammatory responses and autoimmune diseases.

Several cytokines have been shown to be actively involved in metabolic reprogramming in physiological and pathological conditions (312). For example, during cancer cachexia, the overproduction of cytokines significantly increases energy expenditure and leads to weight loss (313). In particular, circulating levels of IL-6 have been shown to positively correlate with cachexia in cancer patients, and importantly, IL-6 levels were found to negatively associate with their survival (314–317). Furthermore, treatment with the humanized anti-IL-6 receptor antibody tocilizumab increased body weight and serum levels of triglycerides and cholesterol in human cancer patients (318). *Il6*-deficient mice have been shown to develop adult-onset obesity with impaired glucose and lipid metabolism (319). The overexpression of IL-6 in high-fat diet-induced obese mice reduced their body weight and improved their obesity-induced fatty liver and insulin resistance (320). Consistently, Arid5a^−/−^ mice showed reduced IL-6 production; mice with long-term loss of Arid5a developed adult-onset severe obesity. In contrast, mice with forced expression of Arid5a are highly resistant to high-fat diet-induced obesity (321). These results suggest that Arid5a is involved in IL-6-mediated metabolic regulation.

**Figure 4:**
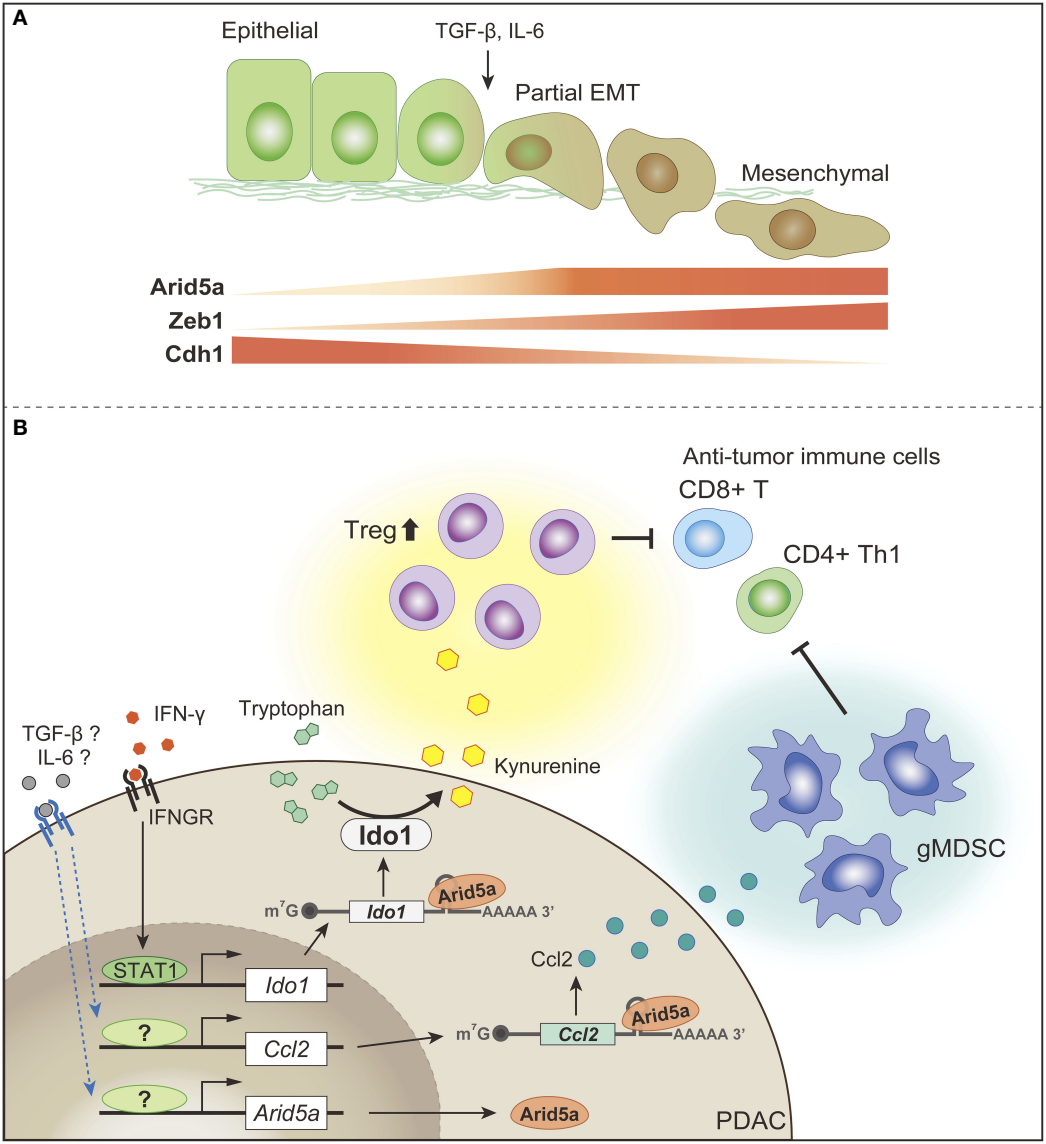
Involvement of Arid5a in the acquisition of mesenchymal plasticity and immune evasiveness in PDAC **(A)** Arid5a expression is associated with acquisition of the mesenchymal phenotypes of PDAC and CRC. Especially, cells showing partial EMT and mesenchymal-like cell lines show much higher expression levels of ARID5A than epithelial-like cell lines. During TGF-β- or IL-6-induced EMT, Arid5a level is augmented in cells that have acquired mesenchymal phenotypes. **(B)** Arid5a acts as a dual regulator in malignant tumors, such as the mesenchymal subtypes of PDAC, to promote an immunosuppressive TME; Arid5a upregulates Ido1 expression *via* post-transcriptional stabilization of its mRNA and then enhances the suppressive effects of Ido1 on anti-tumor immune cells, such as CD8^+^ T cells and CD4^+^ Th1 cells *via* a reduction in intratumoral tryptophan concentration, and a promotion of the differentiation and activation of Treg cells. Additionally, Arid5a post-transcriptionally induces the expression of the chemokine Ccl2 in the TME, which recruits immunosuppressive cells, such as Treg cells and gMDSCs, to the TME.

Recently, we showed that Arid5a mRNA and protein expression levels were significantly increased in mesenchymal tumor subtypes of PDAC and colorectal cancer (CRC), such as quasi-mesenchymal and consensus molecular subtype 4 subtypes, respectively. In addition, Arid5a expression was enhanced in *in vitro* EMT models, induced by IL-6 and TGF-β stimulation (322) (**Figure 4**). Furthermore, Arid5a enables mesenchymal tumor models of PDAC and CRC to facilitate immune evasiveness *via* promoting tumor infiltration of immunosuppressive granulocytic MDSCs (gMDSCs; also known as polymorphonuclear MDSCs (323)) and Tregs (324), and suppressing the recruitment and activation of anti-tumor effector T cells (322). Interestingly, Arid5a acts as a dual regulator leading to the formation of immunosuppressive TMEs in malignant tumors, triggering the metabolic reprograming and recruitment of suppressive immune cells. First, Arid5a induces the inhibitory effect of Ido1 on effector CD4^+^/CD8^+^ T cells *via* the post-transcriptional stabilization of Ido1 mRNA by binding to its 3′-UTR, and a reduction in intratumoral tryptophan concentration (325, 326). Additionally, Ido1 expression in tumor tissues promotes Treg differentiation/activation by generating kynurenine through tryptophan catabolism, and ultimately activating aryl hydrocarbon receptors (AhR) (327, 328), and AhR activation extensively mobilizes gMDSCs (329). Second, Arid5a upregulates chemokine Ccl2 expression in the TME *via* post-transcriptional stabilization of its mRNA, and then Ccl2 leads to enhancement of the infiltration of immunosuppressor cells, such as Tregs and gMDSCs (330–333), to the TME (322).

Therefore, these findings provide insights into the molecular basis of the acquisition of mesenchymal plasticity and immune evasiveness by PDAC and CRC *via* augmentation of the RNA-binding protein Arid5a, and indicate that Arid5a is a promising target for tumor immunotherapy, in addition to inflammatory diseases (**Figure 4**).

## Conclusion and perspectives

In tumorigenesis, metabolic changes and chronic inflammation associated with genetic mutations in normal cells enable transformed cells to escape the homeostatic defense mechanisms of tissues, and to reprogram their intrinsic signaling mechanisms, as well as reprogram populations of stromal cells within the TME and the metabolic balance of the entire organism. In this process, tumor cell populations that adapt to the abnormal microenvironment form diverse, hierarchically organized colonies, and eventually acquire mesenchymal plasticity that promotes their dissemination, reduces the immune system’s ability to counter tumor growth, and finally directly causes death of the organism. Elucidating the metabolic adaptations that tumors rely on to promote these changes and maintain growth in a metabolically unfavorable environment, as well as the molecular mechanisms that trigger the acquisition of mesenchymal plasticity and immune evasion capacity, will help towards developing new diagnostic and therapeutic approaches and dietary combinations for the treatment of PDAC.

Increased levels of IL-6 in the serum have been associated with poor overall survival prognosis in patients with high-grade pancreatic cancer (334), and the increased activity of IL-6/STAT3-mediated signaling has been reported to be associated with poor prognosis in post-resection PDAC patients (335). IL-6 also activates STAT3 and induces the mesenchymal phenotype in human pancreatic cancer cells *via* the induction of SNAI1 (336). In chronic pancreatitis, IL-6/STAT3-mediated ADM transdifferentiation occurs and is associated with PanIN, which is a necessary step for the generation of tumorigenic precursor lesions (220, 337). For example, in the KRAS-induced PDAC mouse model (220, 337), pancreatic epithelial cells with constitutively active KRAS mutations (*KRAS^G12D^*) have been reported to cause inflammatory activation by recruiting immune cells. In particular, myeloid cells have been reported to promote the production of IL-6 and soluble IL6R (sIL6R), activate STAT3 *via* IL-6 trans-signaling, and furthermore, the complex of IL-6 and sIL6R binds to gp130-expressing cells (220). Aberrant STAT3 activation owing to the homozygous loss of *SOCS3* in the pancreas results in the accelerated progression of PanIN and the development of PDAC (220). It has also been shown that KRAS activation increases the levels of cytokines, such as IL-6 and IL-11, in epithelial cells, followed by STAT3 activation in an autocrine manner, and that STAT3-triggered matrix metallopeptidase 7 is required for tumor progression but not tumor development, and may be regulated by other STAT3 targets (337). As already mentioned, the TME of PDAC is severely hypoxic, and nutrient availability is limited by a low vascular density, so PDAC cells show increased autophagy that rewires their metabolism to enable survival in a harsh environment, and to maintain metabolic homeostasis. In a mouse model of PDAC caused by *KRAS* mutations, IL-6-induced STAT3 activation was shown to be involved in the increase in autophagy. As a mechanism, receptors for advanced glycation products have been reported to promote the IL-6-driven activation of STAT3 signaling in mitochondria, bridging autophagy and the IL-6/STAT3 signaling pathway (338). Furthermore, IL-6 signaling has been implicated in the pathogenesis of cachexia in PDAC patients, by inducing a metabolic rewiring (339). Thus, it is clear that the activation of IL-6/STAT3 signaling is involved in the development of PDAC from the PanIN stage, continuing to malignant transformation.

Since its approval in 2009, tocilizumab has been shown to inhibit IL-6/STAT3 signaling in patients with autoimmune diseases, such as rheumatoid arthritis caused by the overexpression of IL-6, acute inflammatory diseases caused by chimeric antigen receptor T-cell therapy, and cytokine storms associated with SARS-CoV-2 infection. On the other hand, in clinical practice, few effective therapeutics have been developed as cancer treatments targeting IL-6/STAT3 signaling (340–343). As mentioned above, cancer is caused by a complex interplay of diverse cell populations, which leads to malignant transformation. Therefore, analysis of the expression and function of molecules associated with IL-6/STAT3 activation may enable the assessment of the local malignant potential and steady state of cancer, but may not be sufficient to predict the stage and detailed course of cancer. Furthermore, it has become clear that not only IL-6/STAT3 signaling, but also various groups of molecules are involved in cancer development. The mode of interaction between these molecules also requires further study.

In the future, it will be essential to introduce spatiotemporal gene expression analysis technology that analyzes multiple cell populations, improve detection technology to analyze the associations among aging, inflammation, and metabolism, and develop artificial intelligence technology to analyze cancer development and progression, and mathematical analysis technology to integrate these technologies. To this end, it is also indispensable to enhance the convergence of life science, physical science, engineering, and computational science to create the next generation of cancer diagnostics and therapeutics.

## Author contributions

AH, HH, and ShH conceived and designed the manuscript. All authors wrote the manuscript, and approved the final manuscript.

## Funding

This work was supported by Grants-in-aid from the Ministry of Education, Science, Sports and Culture of Japan to AH (grant no. 17K08614 and 22K06890) and ShH (grant no. 17K07151 and 22K07203), grants from the Suzuken Memorial Foundation to AH, and Kishimoto foundation to ShH.

## Acknowledgments

We thank H.A. Popiel for critical reading of the manuscript.

## Conflict of interest

The authors declare that the research was conducted in the absence of any commercial or financial relationships that could be construed as a potential conflict of interest.

## Publisher’s note

All claims expressed in this article are solely those of the authors and do not necessarily represent those of their affiliated organizations, or those of the publisher, the editors and the reviewers. Any product that may be evaluated in this article, or claim that may be made by its manufacturer, is not guaranteed or endorsed by the publisher.
